# Synchronous multifocal osteosarcoma with atypical adrenal metastasis: a case report and literature review

**DOI:** 10.1097/MS9.0000000000003383

**Published:** 2025-05-26

**Authors:** Habeeb H. Awwad, Anas K. Assi, Zaina A. Khaled, Haneen Owienah, Mohand Abulilhya, Khaled S. Azzam, Bahaa Bashir, Thaer Sweileh, Feras Eleyan

**Affiliations:** a Faculty of Medicine, Al-Quds University, Jerusalem, Palestine; b Radiology Department, Istishari Arab Hospital, Ramallah, West Bank, Palestine; c Pathology department, Istishari Arab Hospital, Ramallah, West Bank, Palestine; d Oncology department, Istishari Arab Hospital, Ramallah, West Bank, Palestine

**Keywords:** abdominal aortic aneurysm (AAA), adrenal metastasis, lytic lesions, osteosarcoma, synchronous multifocal osteosarcoma (SMOS)

## Abstract

**Introduction and importance:**

The most prevalent malignant bone tumor that typically impacts young adults is osteosarcoma. Synchronous multifocal osteosarcoma (SMOS) with metastasis is extremely rare, defined by the occurrence of various bone lesions being presented at the time of diagnosis. This report describes a rare case of SMOS with adrenal involvement and a simultaneous abdominal aortic aneurysm (AAA).

**Case presentation:**

We report a case of a 34-year-old Palestinian male with a history of hypertension and gout who presented with lower back and right leg pain. Imaging studies showed multiple lytic lesions in the lumbar spine, pelvis, and femur, confirming the presence of a metastatic disease. A whole-body CT scan revealed an adrenal mass and an AAA. A core needle biopsy confirmed the diagnosis of high-grade SMOS with metastasis.

**Clinical discussion:**

Osteosarcoma is the primary cancerous bone tumor that frequently impacts teenagers and young adults. Although multifocal osteosarcoma is rare, it is characterized by its aggressive nature and its possibility to metastasize. SMOS, which is a less common type, makes up only 0.6% of cases. Metastatic SMOS commonly impacts bones, including the pelvis, spine, and femur. While it is typical for cancer to spread to places like the lungs, the rare occurrence of metastasis to organs like the adrenal glands makes diagnosis and treatment more challenging.

**Conclusion:**

This case highlights the challenges in diagnosing and managing SMOS with unusual metastatic patterns and significant comorbidities. It emphasizes the importance of using interdisciplinary, comprehensive methods to optimize patient outcomes in such clinical scenarios.

## Introduction

Osteosarcoma is the most common primary malignant bone tumor, mainly affecting adolescents and young adults, as it is characterized by its aggressive nature and its risk for metastasis^[1]^. However, the multifocal form of this disease is rare, with male predominance^[2]^ and comprising only about 1.5% of all osteosarcoma cases, with the synchronous subtype being rare, comprising only 0.6% of cases^[1]^. Synchronous multifocal osteosarcoma (SMOS) with metastasis is diagnosed with multiple, somehow symmetrical, long bony lesions at the initial presentation, typically within 6 months of symptom onset^[3]^. This aggressive presentation complicates both diagnosis and treatment, as patients may experience widespread skeletal involvement, particularly in the proximal tibia and distal femur^[2]^.

**HIGHLIGHTS**

- Synchronous multifocal osteosarcoma (SMOS) is a rare and aggressive subtype of osteosarcoma, characterized by multiple primary bone lesions presenting simultaneously, often involving weight-bearing bones such as the spine and pelvis, which can severely impact patient mobility and quality of life.
- Unusual metastatic sites, like the adrenal gland, in SMOS are extremely rare and can complicate diagnosis and management, highlighting the need for a thorough and multidisciplinary approach to accurately assess and treat these cases.
- Multidisciplinary care is essential in managing complex oncological cases. Involving oncologists, surgeons, radiologists, pathologists, and palliative care teams early can improve treatment outcomes and provide a holistic approach to the patient’s well-being.

SMOS typically involves weight-bearing bones, such as the pelvis, spine, and femur, which significantly affect patients’ quality of life and mobility. Metastatic spread to other organs, such as the lungs, is common in osteosarcoma^[4]^, but unusual sites of metastasis, like the adrenal glands, have been rarely reported in osteogenic sarcomas, as they need further studies for their diagnosis and treatment^[5]^. Despite advances in chemotherapy and surgical techniques, the prognosis for SMOS remains poor, especially in cases with extensive metastases^[6,7]^.

This case report presents a unique presentation of synchronous multifocal osteosarcoma in a 34-year-old Palestinian male whose clinical presentation was complicated by extensive metastatic disease and significant comorbidities, including an abdominal aortic aneurysm.

The patient first experienced progressive lower back and right leg pain, which revealed in imaging studies multiple lytic lesions across different skeletal sites, including the lumbar spine, pelvis, and femur. The identification of these lesions sparked concerns for metastatic disease, leading to further oncological evaluation. Notably, the presence of an adrenal mass, even though it is non-functional, added difficulty to the diagnostic process and raised doubts regarding the nature of the metastatic spread.

This case highlights the importance of a multidisciplinary approach when dealing with complex oncological presentations, particularly when faced with synchronous multifocal osteosarcoma with metastasis. It underscores the challenges in diagnosing and treating, as well as the need for accurate intervention at the right time. By presenting this case, our goal is to enhance awareness of the atypical presentations of osteosarcoma and the important role of thorough evaluation and assessment in determining effective management approaches. This case report has been reported in line with the SCARE 2023 criteria^[8]^.

## Case presentation

A 34-year-old Palestinian male patient with a past medical history of hypertension and gout was referred to our hospital in August 2024 for neurosurgical and oncological evaluation and management following a lumbosacral spine CT scan that was done in another health facility, which showed multiple lytic lesions scattered at the left head of the femur, acetabulum, right pubis, ischial bone, both iliac bones, and L2, L3, and L5 vertebral bodies associated with a pathological fracture of the upper endplate of the L2 vertebra. Initially the patient complained of progressive lower back and right leg pain; he described the pain as constant, limiting his mobility, and worsening with activity for several months, with no history of trauma. On physical examination, there was a localized tenderness over the lumbar spine and pelvic region, and a palpable mass was noted in the right groin; no neurological deficits were detected.

**Figure 1.**
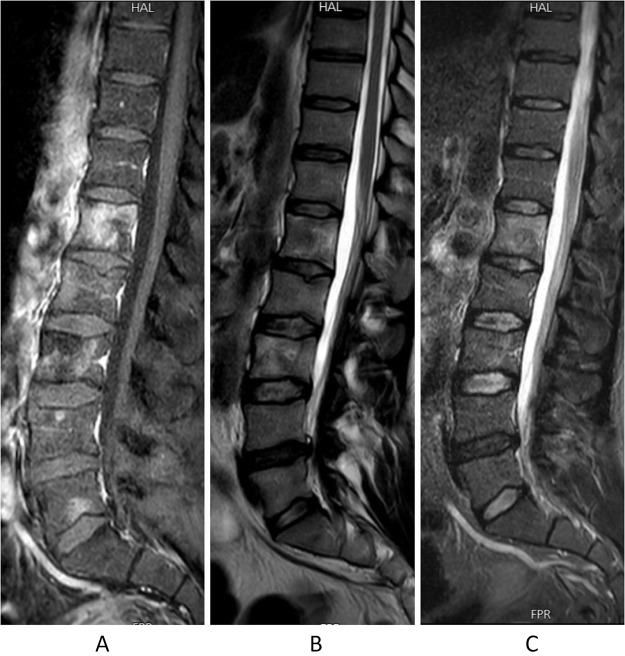
Lumber spine MRI with IV contrast was done and showed the following (blue arrows): (A) sagittal T1 weighted image with IV contrast, (B) sagittal T2-weighted image, and (C) sagittal short tau inversion recovery (STIR). There are multiple heterogeneously enhancing vertebral body lesions with variable signal intensity and high T2WI and short tau inversion recovery (STIR) sequences. There is a cortical disruption in the upper end plate of L2 vertebral body, with no significant decrease in height. L4–L5 level shows dehydrated disc with posterior disc bulge compressing the thecal sac. No significant spinal canal narrowing.

On 21 August 2024, a whole spine MRI with IV contrast was done (Fig. 1) and showed multiple heterogeneously enhancing vertebral body lesions with varying signal intensity, primarily involving the lumbar spine, right L3 pedicle, sacrum, and iliac bones. These lesions were suggestive of metastatic bone disease. Cortical disruption of the L2 vertebral body was noted, though there was no significant loss of height. Noticeably, soft tissue lesions were noted encasing the abdominal aorta and in the left adrenal region, requiring further evaluation.

**Figure 2.**
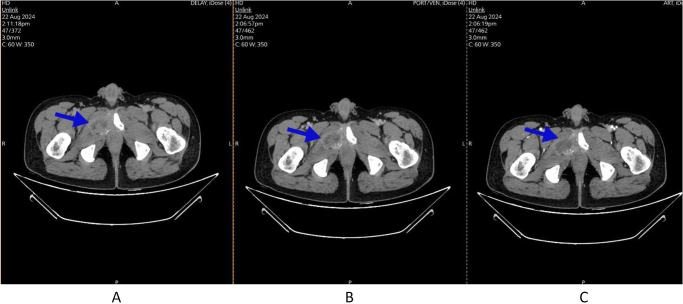
Axial views of CT scan with IV contrast for pelvic region was done and showed the following: a lytic lesion in right inferior pubic bone showed soft tissue component, with heterogeneous progressive enhancement post contrast administration (blue arrows): (A) delayed phase, (B) venous phase, and (C) arterial phase. A core needle biopsy was obtained from this lesion.

**Figure 3.**
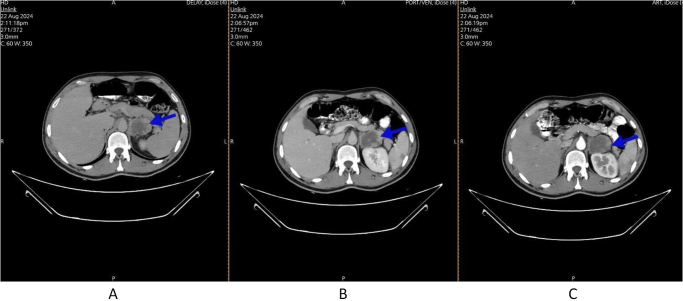
Axial view of CT scan with IV contrast was done and showed the following: a Left adrenal mass measuring about (4.8 × 4 cm), with heterogeneous progressive enhancement post contrast administration (blue arrows): (A) delayed phase, (B) venous phase, and (C) arterial phase.

**Figure 4.**
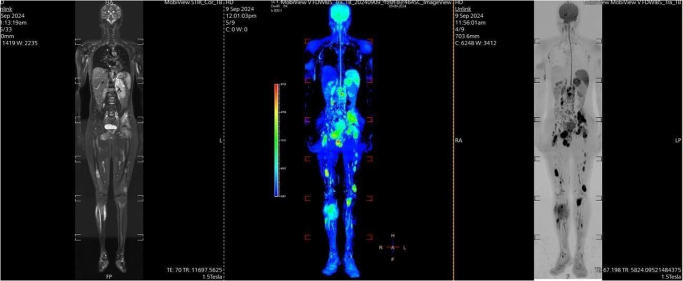
A whole-body diffusion weighted MRI shows multiple lesions in the spine, pelvic bones and lower limb skeleton.

On 22 August 2024, a whole-body oncology CT scan with contrast (Figs. 2 and 3) and a whole-body diffusion-weighted MRI were obtained (Fig. 4). Multiple lytic lesions were seen in the lumbar and sacral spine, pelvic bone, and both lower limb skeletons (Fig. 4). A lytic lesion in the right inferior pubic bone showed a soft tissue component (Fig. 2). These findings were consistent with extensive metastatic disease. It also showed a left adrenal mass measuring about (4.8 × 4) cm, with heterogeneous progressive enhancement post-contrast administration (Fig. 3). There was a (1.5 × 1.8 cm) lesion seen in the left external obturator muscle, mostly representing metastatic deposits. Even though this is an unusual pattern of metastasis, both the left adrenal mass and the lytic lesion in the right inferior pubic bone share the same radiological features, including a similar enhancement pattern and diffusion restriction on whole-body diffusion-weighted imaging, which further suggests a metastatic process.

A focal aneurysmal dilation with mural thrombosis of the infrarenal abdominal aorta, measuring (4.6 × 5.8 × 5.5 cm), was noted. A vascular surgery team was consulted, and their impression was that the patient may have had a previous rupture and hematoma, as CT showed, so the patient needs to undergo endovascular aneurysm repair (EVAR) based on his prognosis that was not yet confirmed. So, the patient was started on a therapeutic dose of anticoagulation.

The endocrine team was consulted regarding the adrenal mass seen on CT. Adrenal function tests were ordered; results were normal, and their impression was that the mass is nonfunctional. Adrenal mass biopsy was not done due to patient refusal.

**Figure 5.**
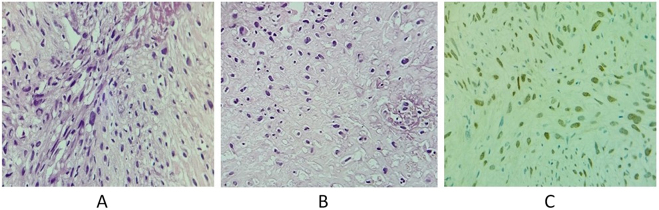
Histologic sample shows: (A) highly atypical and pleomorphic tumor cells with frequent mitotic figures (H&E, 40×), (B) some areas show osteoid deposition, necrosis is seen (H&E, 40×), and (c) SATB2 immunohistochemical stain. H&E: hematoxylin and eosin. SATB2: special AT-rich sequence binding protein 2.

On 24 August 2024, a core needle biopsy of the right groin mass was performed to determine the origin of the tumor and was sent to King Hussein Cancer Center for consultation. The report came back on 5 September 2024, showing a high-grade malignant mesenchymal neoplasm, consistent with osteosarcoma. Tumor cells were highly atypical and pleomorphic, with frequent mitotic figures and areas of necrosis. Some areas showed osteoid deposition. Immunohistochemistry stains were negative for Cytokeratin 7, Cytokeratin 20, Desmin, CD34, double-minute type 2 (MDM2), SRY-related HMG-box 10, Erythroblast transformation-specific-related gene, Synaptophysin, S-100, Sal-like protein 4, CD31, and Pancytokeratin, which were all negative. But the smooth muscle actin and the special AT-rich sequence-binding protein 2 (SATB-2) were focally positive, so this supported the diagnosis of osteosarcoma despite the pattern of metastasis, which was unusual (Fig. 5).

On 31 August 2024, the patient underwent a transesophageal echocardiogram, which was done to rule out malignancy-related endocarditis, and the results were unremarkable. Two hours later, the patient developed sepsis. Blood cultures were done and showed polymicrobial infection. The infectious disease team recommended treating the patient with colistin (polymyxin E) for 7 days due to the multidrug-resistant organisms. The infection resolved after appropriate antibiotic therapy.

On 7 September 2024, a multidisciplinary team meeting (MDT) was held to discuss the best approach, involving oncologists, vascular surgeons, and radiologists. A final diagnosis of synchronous multifocal osteosarcoma (SMOS) with metastasis was made. MDT recommended starting the patient on Cisplatin and Adriamycin chemotherapy over three consecutive days. The initial dose was delayed due to a febrile episode, but there were no significant adverse reactions during the treatment.

On 16 September 2024, the patient underwent EVAR for the infrarenal abdominal aortic aneurysm (AAA), which was successfully repaired using bilateral femoral access. Multiple stent grafts were deployed, and balloon dilation ensured proper placement. There were no postoperative complications.

During recent admissions, the patient’s state deteriorated, and multisystem complications developed due to aggressive, chemotherapy-resistant disease. Despite two lines of chemotherapy (cisplatin/doxorubicin followed by gemcitabine/docetaxel) and pelvic radiotherapy, imaging confirms disease progression, including persistent right iliac lesions. Vascular complications also developed, such as superior mesenteric artery stenosis requiring stenting and celiac trunk occlusion that heightened the risks of mesenteric ischemia and thrombosis. Concurrent gastrointestinal issues, including bowel dilatation related to malignancy and melena, compound his frailty. And most recently, persistent thrombocytopenia and anemia limited his chemotherapy and anticoagulation (Clexane). While cachexia, recurrent infections, and transfusion dependency reflect end-stage decline, survival is likely limited with escalating risks of bowel perforation, sepsis, or vascular catastrophe, necessitating prioritization of palliative care for symptom control and ethical reassessment of futile interventions.

The patient initially received first-line chemotherapy with cisplatin and Adriamycin. Then, a second-line gemcitabine and docetaxel were introduced. Both were delayed due to thrombocytopenia. Pelvic radiotherapy and bisphosphonates were added for local tumor control and bone metastases. Procedural interventions included EVAR for AAA repair and angioplasty for celiac occlusion. Supportive care involved recurrent PRBC transfusions, adjusted Clexane dosing to balance thrombosis and bleeding risks, opioids (Targin/Percocet) for pain, electrolyte repletion, and bowel management with enemas. While polymicrobial sepsis was treated with colistin, isolation protocols were maintained for rectal CRE colonization.

## Discussion

There are two types of multifocal osteosarcoma (MFOS): metachronous, which is a new tumor that develops after the initial therapy, usually over a period of 6 months, and synchronous, which is more than one lesion at presentation and often develops within 6 months. Just 1.5% of all osteosarcomas were MFOS, with 0.9% and 0.6% of cases of each kind, respectively. Amstutz HC6 defined multifocal osteosarcoma in 1969 as a condition in which many skeletal lesions coexist with a single main tumor. He described two forms: a synchronous form and a metachronous form^[1]^. In addition, the prognosis of multifocal osteosarcoma is reportedly poor, especially in metachronous cases compared to synchronous ones^[9]^.

The most prevalent primary malignant tumor of bone that affects young adults and mostly affects the long bones is called osteosarcoma. It has a bimodal distribution of ages, ranging from young to old. Between the ages of 10 and 14, there is the first discernible peak, and in people over 65, there is the second^[10]^. While MFOS mainly affects young patients, with a mean age of 16, there is a male predominance. Osteosarcomas that are metachronous and those that are synchronous do not substantially differ in terms of clinical features or involvement site^[2]^. Here in our case, the presence of multiple lytic lesions in the spine, pelvis, and femur is characteristic of SMOS with metastasis. The involvement of weight-bearing bones, particularly with a pathological fracture of the L2 vertebra, significantly impacts the patient’s mobility and quality of life.

The lung is the most common extraosseous site to be involved in osteosarcoma with a poor prognosis^[11]^. On the other hand, many studies of multifocal osteosarcoma have shown an absence of metastasis to the lung, which rules out hematogenous spread^[5]^. While adrenal gland metastasis is almost common in the cases of malignant epithelial tumors, as they are discovered in 27% of individuals with these types of tumors. Adrenal metastasis from bone tumors is extremely rare, and even more so for osteogenic sarcomas^[7]^. As unusual, an adrenal mass was discovered in our patient on imaging that raised concerns for metastatic spread, given the patient’s aggressive osteosarcoma.

In general, understanding the exact mechanisms of metastasis progression of osteosarcoma is still required because it’s still not clear enough, despite the recent advancements in prediction and early detection of metastasis. Therefore, the most important mechanisms of metastasis of osteosarcoma include, first, the hematogenous spread, which is the most common, where osteosarcoma spreads through the bloodstream. Second, the lymphatic spread, which is less common in osteosarcoma, occurs when tumors reach the adrenal gland via retroperitoneal lymphatic pathways. Third, the direct extension, which is very unlikely and extremely rare^[12]^. Given the limited number of such documented cases, adrenal metastases are considered extremely rare and thought to be mainly spread through hematogenous spread^[12]^.

SMOS is a rare condition, with a reported incidence of 1 to 3%^[13]^. There are many discussions in literature about whether SMOS represents multiple primary tumors or metastatic disease. The majority of studies come to the conclusion that SMOS represents one extreme of a wide spectrum of metastatic osteosarcoma^[5]^. SMOS manifests as a single dominant lesion of a primary osteosarcoma, with the remaining lesions suggesting metastasis. Typically, these lesions have a narrow transition zone, are purely sclerotic, and do not exhibit any signs of cortical destruction, soft-tissue mass, or malignant periosteal new bone formation^[5]^.

In general, CT scans and MRIs were the primary imaging studies used for osteosarcoma diagnosis and staging. These two techniques were superior in determining the original lesion’s anatomical extent^[1]^. In the past, when chemotherapy was started to be used, SMOS with metastasis was thought to be fatal in a few months. Despite that, combined surgery and chemotherapy significantly raised the treatment success rate to 60–70% for common osteosarcoma without visible metastases^[1]^. Unfortunately, the large infrarenal abdominal aortic aneurysm with mural thrombosis added complexity to the case. This aneurysm required repair, so EVAR was successfully performed, stabilizing the vascular complication. In addition, the patient’s sepsis, secondary to polymicrobial organisms, delayed chemotherapy initiation. However, prompt detection and treatment with appropriate antibiotics allowed rapid recovery, and then the patient accomplished his first cycle of Cisplatin and Adriamycin chemotherapy.

Moreover, the integration of radiological findings and histopathological analysis further assists in the diagnosis of SMOS in this patient and clarifies its atypical presentation and guides clinical decisions. Imaging revealed multiple lytic lesions that correlated directly with histopathological evidence of osteoid deposition, a hallmark of osteosarcoma^[14]^. Radiologically, heterogeneously enhancing masses were identified in the left adrenal gland and pelvic obturator muscle, raising concerns for metastatic carcinoma due to their unusual locations. However, immunohistochemical analysis provided clarity in which focal positivity for SATB-2 confirmed osteoblastic differentiation. Negative markers for epithelial (CK7/CK20), melanocytic (S100), and vascular (CD31) tumors excluded other mimics such as Ewing sarcoma or dedifferentiated liposarcoma (negative for MDM2 amplification) or primary adrenal malignancies (negative for neuroendocrine markers like Synaptophysin)^[15]^. This integration of radiological and pathological findings solidified osteosarcoma as the diagnosis despite the atypical metastatic pattern.

The adrenal lesion was not biopsied due to patient refusal. Therefore, the diagnosis relied on radiological and clinical findings, which strongly supported metastatic involvement. The left adrenal mass demonstrated heterogeneous, progressive post-contrast enhancement without evidence of lipid-rich content – features inconsistent with benign adrenal adenomas, which are typically smaller, homogeneous, and exhibit rapid contrast washout with low attenuation on non-contrast CT. Primary adrenal malignancy was unlikely due to the absence of calcifications, necrosis, or markedly irregular margins, along with no clinical or historical indicators supporting a primary adrenal origin. Functional adrenal tumors such as pheochromocytoma were excluded based on normal catecholamine and metanephrine levels, as well as the absence of characteristic symptoms. Infectious or hemorrhagic causes were also considered unlikely due to the lack of fever, elevated inflammatory markers, trauma history, or imaging features such as rim enhancement or hemorrhagic signal changes. Noticeably, the adrenal lesion shared key radiological features with the confirmed metastatic lesion in the right inferior pubic bone, including a similar enhancement pattern and diffusion restriction on whole-body diffusion-weighted imaging. These findings, in combination with the lesion’s size, imaging behavior, and the context of widespread metastatic skeletal disease, provide strong support for a metastatic origin.

The management of this case faced numerous interconnected challenges that significantly impacted decision-making. The diagnostic complexity resulted from the disease’s atypical presentation and unusual metastatic pattern. This diagnostic uncertainty was compounded by the patient’s refusal to obtain an adrenal biopsy, which hindered timely treatment initiation. That necessitated extensive multidisciplinary coordination, involving oncology, radiology, pathology, vascular surgery, endocrinology, and infectious disease teams. Treatment decisions were further complicated by the need to balance potential benefits against significant risks in a patient with deteriorating health. The aggressive nature of SMOS demanded systemic chemotherapy, but persistent thrombocytopenia and anemia delayed treatment regimens. At the same time, the medical team had to decide whether to pursue aggressive interventions or switch to palliative care, which was a difficult moral decision. As the disease progressed, escalating systemic complications further restricted therapeutic options, underscoring the need for continual reassessment of the care plan.

## Conclusion

This case represented an atypical site of metastasis for SMOS and showed an unusual course of the disease, which appeared in an aggressive presentation with more than five lytic lesions. In addition, the site of metastasis, which mainly appeared in the adrenal gland, was extremely rare, as mentioned before. Finally, our case highlighted the challenges in identifying and managing SMOS with metastasis, especially the notable comorbidities the patient suffered from. This illustrates how crucial it is to use comprehensive, interdisciplinary approaches to enhance patients’ outcomes in such difficult medical situations.

## Data Availability

None.

## References

[1] Setiawati R Lay ES Testini V. Advance MR evaluation of synchronous multifocal osteosarcoma with pathologic fracture. BJR Case Rep 2021;7:20210015. 35047204 10.1259/bjrcr.20210015 PMC8749407

[2] Bulut HI Kanay E Anarat FB. Synchronous multifocal osteosarcoma: report of 4 cases and literature review. Surg Case Rep 2024;3:100062.

[3] Qin LF Fang H Qin LH. Synchronous multifocal osteosarcoma with hypocalcemia. Libyan J Med 2013;8. 10.3402/ljm.v8i0.20359 PMC3585775 23463848

[4] Odri GA Tchicaya-Bouanga J Yoon DJY. Metastatic progression of osteosarcomas: a review of current knowledge of environmental versus oncogenic drivers. Cancers (Basel) 2022;14:360. 35053522 10.3390/cancers14020360 PMC8774233

[5] Agrawal M Patil A James T. Multifocal osteosarcoma: multiple primaries or metastases? A report of rare case and review of literature. J Orthop Case Rep 2020;10:97–100. 10.13107/jocr.2020.v10.i08.1878 PMC7933644 33708722

[6] Bhutani M Pathak AK Sengar M. Multifocal osteosarcoma involving unusual sites. Cancer Invest 2006;24:278–82. 16809155 10.1080/07357900600629559

[7] Siddiqui NH Jani J. Osteosarcoma metastatic to adrenal gland diagnosed by fine-needle aspiration. Diagn Cytopathol 2005;33:201–04. 16078243 10.1002/dc.20335

[8] Sohrabi C Mathew G Maria N. The SCARE 2023 guideline: updating consensus Surgical CAse REport (SCARE) guidelines. Int J Surg Lond Engl 2023;109:1136. 10.1097/JS9.0000000000000373 PMC10389401 37013953

[9] Kusakabe M Sakamoto A Shimizu T. Paraplegia caused by multifocal osteosarcoma with spinal lesions. Int J Spine Surg 2021;15:1234–37. 35086882 10.14444/8156 PMC9541608

[10] Astien A Hairunisa N Abikusno N Abikusno R Agil N. Metachronous multifocal osteosarcoma after 5-month therapy: metastasis or other primary lesion? Jurnal Biomedika Dan Kesehatan 2023;6:347–53.

[11] Huang X Zhao J Bai J. Risk and clinicopathological features of osteosarcoma metastasis to the lung: a population-based study. J Bone Oncol 2019;16:100230. 30923668 10.1016/j.jbo.2019.100230 PMC6423404

[12] Basu S Shet T Awasare S. Bilateral adrenal metastases and metastatic subcutaneous deposit in the chest wall from osteosarcoma of the mandible: utility of 18F-FDG-PET. Hell J Nucl Med 2009;12:51–54. 19330184

[13] Sato H Hayashi N Yamamoto H. Synchronous multifocal osteosarcoma involving the skull presenting with intracranial hemorrhage – case report. Neurol Med Chir (Tokyo) 2010;50:407–09. 20505300 10.2176/nmc.50.407

[14] Bacci G Fabbri N Balladelli A. Treatment and prognosis for synchronous multifocal osteosarcoma in 42 patients. J Bone Joint Surg Br 2006;88:1071–75. 16877608 10.1302/0301-620X.88B8.17809

[15] Gon S Kundu T Ghosh B. Synchronous multifocal osteosarcoma with small cell histological variant: a double rarity. Clin Cancer Investig J 2016;5:533–36.

